# Assessing Esports Participation Intention: The Development and Psychometric Properties of the Theory of Planned Behavior-Based Esports Intention Questionnaire (TPB-Esport-Q)

**DOI:** 10.3390/ijerph182312653

**Published:** 2021-11-30

**Authors:** Ka-Man Leung, Ming-Yu-Claudia Wong, Kai-Ling Ou, Pak-Kwong Chung, Ka-Lai Lau

**Affiliations:** 1 Department of Health and Physical Education, Hong Kong University of Education, Hong Kong; leungkaman@eduhk.hk; 2 Department of Sport, Physical Education and Health, Hong Kong Baptist University, Kowloon, Hong Kong; 21482268@life.hkbu.edu.hk (K.-L.O.); pkchung@hkbu.edu.hk (P.-K.C.); 18482120@life.hkbu.edu.hk (K.-L.L.)

**Keywords:** Esports, theory of planned behavior, psychometric properties, Esports participation

## Abstract

Background: Esports is seen as an emerging industry that has enjoyed a surge in popularity worldwide. As a result, researchers have undertaken studies to try to understand the motivations and factors that impact Esports gameplay. Given the extensive utilization of TPB in many research projects to conceptualize and predict various behaviors, the current study aimed to further extend this theory to the Esports context by developing and validating an instrument that can illustrate the factors that impact the intention to participate in Esports, thus predicting Esports game playing behaviors. Methods: A total of 25 participants were involved in the development of the questionnaire using the qualitative approach, while 915 university students and 1164 secondary school students were involved in the survey for the questionnaire validation using the exploratory factor analysis and the confirmatory factor analysis. Results: All measurement models of the TPB subscales are considered as good fit. Conclusion: Hence, showing the newly designed TPB Esports Intention Questionnaire was found to be reliable and valid in revealing the level of intentions as well as the factors affecting Hong Kong students playing Esports.

## 1. Introduction

Esports (electronic sports or e-sports) is “competitive (pro and amateur) video gaming that is often coordinated by different leagues, ladders and tournaments, where players customarily belong to teams or other sporting organizations, which in turn are sponsored by various business organizations” [1]. In recent years, Esports has been seen as an emerging industry that has enjoyed a surge in popularity worldwide. Currently, it has a large following that rivals those of other major professional sports, such as the National Basketball Association (NBA) and National Football League (NFL). A report from investment firm Goldman Sachs (2018) estimated that the global monthly audience of Esports at that time was about 167 million, and it would grow to 276 million by 2022. In terms of modernization, the industry generated USD 869 million in 2018, and the firm forecasted that the number would grow to USD 2.96 billion in 2022 [2]. These numbers illustrated the enormous potential of Esports, which can be considered extending beyond watching Esports competitions or consuming Esports through the various media platform. In fact, the current size of the Esports audience base can be regarded as a critical indicator of Esports participation. Before we explore this notion further, we will define Esports participation in the context of this research. In this research, Esports’ participation is referred to as those who engaged in playing electronic games competitively with spectators or audience, while the games should be structurally organized. The participants were also expected to engage in regular training, with pre-planned tactics, skills, and strategies, just like traditional sports’ training and competition. Under this context, early research has indicated that there is a relationship between gaming video consumption (watching), video game playing, and money spending behaviors. The study found that the three forms of gaming video content (game reviews, let’s play, and Esports) have a positive relationship with time spent on playing video games [3], and gaming video consumption is also positively linked to game purchasing behavior. In another study that investigated how watching Esports impacts game consumption (both playing and spending money on games), the researchers also found there is a strong relationship between Esports spectating and gaming intention and consumption [4]. These findings suggest within the context of Esports, watching Esports has a significant impact on Esports gameplay and spending on games.

The research on the relationship goes beyond the relationship between Esports consumption and Esports gameplay. There is also a focus on the factors that motivate Esports gameplay. Early research in this area from Lee and Schoenstet [5] indicated that competition factor and skill factor had a significant impact on the time spent on Esports gaming when peer pressure had a marginal significance. Similarly, Billings, Lewis, and Bissell [4,6] adopted previous studies on motivations to play in traditional sports and fantasy sports to measure different motivations that are relevant to Esports gameplay. Those motivations included arousal, camaraderie, competition, escape, pastime, self-esteem, social sport, fanship, and Schwabism. The researchers found competition, passing time, and Schwabism had a significant, direct relationship to the number of hours engaged in Esports gameplay participation. Furthermore, the researchers also revealed that camaraderie, arousal, fanship, and Schwabism had a significant, direct relationship with the Esports participants’ future intent to participate in Esports.

More recently, researchers have started to utilize theories from other disciplines and develop models that explain Esports gameplay behavior. For instance, Jang and Byon (2019) employed the Unified Theory of Acceptance and Use of Technology 2 (UTAUT2) from the information technology field, to explore the antecedents and consequences related to Esports gameplay. Building upon the UTAUT2, the researchers proposed the Esports Consumption (ESC) model, which consists of six determinants that impact the intention to engage in Esports gameplay (hedonic motivation, habit, price value, perceived effort expectancy, social influence, and flow), and a behavioral consequence (intention to consume media related to Esports events. After analyzing the data that was collected from two different times (six months apart), the researchers revealed that hedonic motivation, price value, effort expectancy, and flow were crucial in influencing individuals’ intention for Esports gameplay. Additionally, the researchers also confirmed there was a sequential relationship among the intention to engage Esports gameplay, actual Esports gameplay, and intention to consume media of Esports events.

In line with this research, the current study aims to extend the work in this area, utilizing a notable social behavior theory to understand the attitude toward Esports and to predict its gameplay. While much of the previous Esports research was conducted in regions with high participation rates, the current study tries to understand Esports gameplay behavior from the perspective of a region with low participation rate. It is sensible to believe that a region with low participation will be conducive to understanding the potential barriers to Esports gameplay. One of the dimensions (behavioral control in the theory of planned behavior) in the study’s theoretical framework is designed to explore this very issue.

The region being examined is the city of Hong Kong. Given the global popularity of Esports, Hong Kong Government has attempted to proactively boost the local gaming industry and promote Esports as “a new sector with economic development potential” industry. Yet, there was an intensive debate in the public about the possible development of Esports in Hong Kong, as well as the potential impact of Esports on the health and education of youngsters. The lack of understanding in the nature of Esports has caused hesitancy among the potential players, as it was revealed in a local Esports survey [7] that only 13.4% of the 1400 survey participants participated in Esports competitions, and the reasons for the low participation rate were mainly caused by the restriction of parents and the negative social image of playing video games. The research team [8] has also completed qualitative research and indicated that Hong Kong has no full-time Esports athletes; and Hong Kong young adults’ Esports participation was mainly affected by the negative social perception towards Esports, the immature career development of Esports in Hong Kong and the lack of financial support for athletes. Owing to the above, the non-commercialized culture of the Hong Kong sports context might have hindered the development of Hong Kong Esports, thus affecting the Esports’ participation of the Hong Kong people.

Therefore, to tap into these new Esports markets more efficiently, it is crucial to understand the local communities’ attitudes toward Esports, their participation (playing) intention, and the factors contributing to their attitudes. Given this backdrop, this paper will present the development process of an instrument that utilizes the theory of planned behavior (TPB) [9] as the theoretical framework, with the aim of gauging the attitude and behavioral intention to participate (playing) in Esports gaming and competition. In addition, the psychometric properties of the instrument will also be reviewed to ensure that it is sufficiently reliable for use in future Esports consumption studies, both in the academic setting and in the field.

TPB is one of the most prominent theories in predicting social behavior, evolving from Ajzen and Fishbein’s earlier work on the theory of reasoned action (TRA) [10,11]. TRA postulates that the behaviors of individuals who act rationally will be predicated on behavioral intention. In turn, behavioral intention is influenced by two factors: attitudes toward behavior and subjective norms. The researchers considered attitude as reflecting an evaluation of the probability of undertaking a specific behavior that would lead to a preferred outcome, rooted in personal beliefs and salient information available [12]. Subjective norm refers to the individuals’ perceived opinions of their significant others on their engagement in the said behavior [13]. Subsequently, Ajzen recognized that the variance of the TRA model [9] in predicting behavior was limited by the specified condition of pure volitional control, and thus implemented the third factor, perceived behavioral control, to address this shortcoming, thus establishing TPB. Within the framework of TPB, perceived behavioral control denotes individuals’ perceptions of their abilities, resources, and opportunities to perform said behavior [12].

Given the extensive utilization of TPB in many research projects to conceptualize and predict various behaviors, ranging from volunteer behaviors [14,15] to health behaviors [16,17], online shopping [18], and sporting event attendance [7,19], it is reasonable to suggest that the theory’s generalizability has been substantiated. In the current study, we plan to further extend this theory to the Esports context by developing and validating an instrument that can illustrate the factors that impact the intention to participate in Esports, thus predicting Esports behaviors.

**Hypothesis** **1** **(H1).** *The Psychometric Properties of the Theory of Planned Behavior-Based Esports Intention Questionnaire (TPB-Esport-Q) is examined to be reliable and valid*.

## 2. Study 1

The purpose of Study 1 was to develop an item pool and develop initial measurement items to capture participants’ indirect measures of sports participation under each TPB construct.

### 2.1. Method

The TPB Esports Intention Questionnaire was structured using standardized questions that tap into the constructs of TPB to measure participants’ attitudes, subjective norms, and perceived behavioral control, with indirect measures suggested by Ajzen [20].

The questionnaire was scored on a 5-point Likert Scale, and the items of the direct measures included behavior (one item), intention to participate in Esports in the coming six months (three items), attitude towards Esports participation (four items), subjective norms about participating in Esports (four items), and perceived behavioral control of participating in Esports (four items). Example items of behavior and subjective norm are: “In the coming 6 months, I will participate in Esports competitions” and “I feel under social pressure to participate in Esports competitions”.

The indirect measures’ items were not standardized by TPB Measurement Constructs; therefore, with reference to suggested guidelines [21], 25 students, stratified by (i) secondary and university students and (ii) having participated in Esports competitions in the last 12 months, were recruited to participate in focus group interviews (five per group). At the interview, the participants were asked open-ended questions about their (1) views of Esports; (2) experiences in Esports; (3) attitudes toward Esports participation, such as positive and negative aspects; (4) views about the influences of Esports on students; (5) significant others who support or do not support their participation in Esports; and (6) enablers or barriers to Esports participation. The interview guide was developed according to guidelines suggested by Fishbein and Ajzen [22] based on the TPB construct.

Due to COVID-19, both face-to-face and online focus group interviews were offered as options for the participants. Two onsite groups of interviews were conducted in the meeting room of our partner company, and the remaining three were conducted via a Zoom meeting. Before the focus group interview, participants were informed of the objectives of the study and their right to leave the project at any time, and that their personal data would be kept confidential and destroyed after the completion of this research. Interviewees who had participated in online interviews were requested to move to a quiet place with access to a stable-speed network before the interview began. Three investigators (KLO, MYW, and LKM) who were familiar with the purpose of the study were involved in moderating focus group discussions. They were female, knowledgeable about focus groups and Esports, and had no prior relationships with the interviewees. All interviews were audiotaped. During the interviews, some observational notes were recorded by the investigators to enhance the accuracy of the transcripts and data analysis. Participants had the right to view the transcripts. After the completion of the focus group interviews, participants were given an HKD100 supermarket voucher for their contribution to our study. The interviews took an average of 65.2 min (range = 42–94 min) to complete.

### 2.2. Participants

Twenty-five teenagers and young adults participated in the study. Teenagers and young adults were defined as individuals studying in secondary schools or universities (aged 15–29 years) in Hong Kong. This age range was chosen based on previous studies [7,23] that found that most of the population playing Esports and video games were aged between 15 and 29 years (studying in secondary schools or universities). Among the 25 participants (24 males), 52% were university students, and their mean age was 18.72 years (range = 15–26 years). All participants had participated in Esports competitions in the last 12 months. Regarding gaming type, Table 1 shows that the most popular game among participants was League of Legends (LOL; 64%), followed by PlayerUnknown’s Battlegrounds (PUBG mobile; 44%).

### 2.3. Results

The transcripts were qualitatively analyzed through thematic content analysis using NVIVO 12, and analytic induction was used to describe the participants’ perceptions [24]. Based on the analytic coding, several categories from each indirect measure component were established, including beneficial and deleterious outcomes (goal setting and achievement, physical health, socialization and teamwork, psychological benefits, academics and time distribution, physical strain, and negative social image and perception toward Esports participation), subjective norms (parents, peers, teachers, and people with particular characteristics and reference), and barriers and facilitators (balance between academics and Esports, capability, career prospects and future reality, psychological benefits, and peer encouragement and support). These categories were all considered as initial indirect measure items.

Based on the focus group interview results, the initial Esports Participation Questionnaire was developed, comprising 13 standardized direct measures items and 50 indirect measurement items (see Supplementary Materials for the initial Esports Participation Questionnaire).

## 3. Study 2

The aims of Study 2 were to (1) confirm the factor structure of the initial TPB Esports Intention Questionnaire, (2) examine the reliability and validity of the TPB Esports Intention Questionnaire, and (3) compare the differences between secondary school and university students in terms of insignificant items and measurement invariance.

### 3.1. Method

#### 3.1.1. Participants

The population of this study included students studying in secondary schools and universities. Secondary school students were aged 15–20 years studying in F4 to F6, while university students were full-time students aged 29 years or below and studying in diploma, sub-degree, or degree programs in UGC-funded universities in Hong Kong. This age range was selected with reference to previous studies [7,25] that found that the major population of Esport players were aged 15–29 years old. Other inclusion criteria for participants were permanent residency in Hong Kong and literacy in Cantonese.

A total sample size of 2115 students participated in the survey, which is an adequate sample size for an estimated parameter (at least 10:1 ratio) for confirmatory factor analysis [26]. In addition, this sample size meets the guidelines for conducting a population-based survey suggested by the World Health Organization [27], which is within 2% of the reported true population of sports participation (13.4%) [7].

#### 3.1.2. Procedures

The study period was from March to September 2020. First, the newly developed questionnaire was subjected to a pilot test with five university students (three participants and two non-participants) and two Esports experts from ER Esports (company name) to evaluate the questionnaire’s length, wording, and formatting. Before data collection, an invitation letter was sent to a randomly selected secondary school. A paper and a web-based questionnaire with consent form were created for schools to choose from. Due to COVID-19, there was no positive response from the selected schools, so convenience sampling was applied to reach other schools in the districts. With the principals’ consent, a trained research assistant sent web-based questionnaires or handed out paper questionnaires for data collection to the responsible teachers.

For university students, an invitation e-poster attached to a QR code for the web-based questionnaire was sent to students email via mass email. At schools for which we were unable to obtain a student mailing list, the e-poster was sent to the student affairs office or physical education teachers at the school for promotion. All participants completed the questionnaire only once, and the average completion time was 10 min. Prior to administering the questionnaires, permission was granted by the Institutional Review Board for Research with Human Subjects of the University. After collecting data, all participants were randomly drawn, and 50 winning students received an HKD 500 supermarket voucher. Participants were informed of the confidentiality of the data and were free to discontinue their participation in this study.

#### 3.1.3. Measures

Participants were asked to respond to questions about their demographic information, including their age, gender, education level, school, house type, and family income. Based on the theory of planned behavior (TPB), the Esports Intention Questionnaire was developed in Study 1, mainly to explore Hong Kong teenagers and young adults’ Esports participation and their respective attitudes toward the sport.

Apart from the standardized questions for direct measures, the indirect measure items were developed based on the results of Study 1. Behavioral beliefs included Hong Kong teenagers’ views on the beneficial and deleterious outcomes (Goal Setting and Achievement, Physical Health, Socialization and Teamwork, Psychological Benefits, Academics and Time Distribution, Physical Strain, Negative Social Image, and Perception of Esport Participation), for example, “My participation in Esports in the next six months will help my life goal setting” and “Setting my life goal is extremely bad … extremely good”. Normative beliefs included participants’ perceived social pressure or standard form (Parents, Peers, Teachers and People with particular characteristics and reference), for example, “Whether my parents and families think I should participate in Esports or not in the next months” and “When it comes to Esports participation, how much do you want to do what your family thinks you should do?” Control beliefs concern barriers and facilitators (Balance between Academic and Esports, Capability, Career Prospects and Future Reality, Psychological Benefits, and Peer Encouragement and Support) for participation in Esports; example questions are: “I expect that participating in Esports in the next six months will disrupt my academics” and “My academic disruptions in the next six months would make it easier for me to quit Esport participation”.

#### 3.1.4. Statistical Analyses

Data were analyzed using the Statistical Package for the Social Sciences (SPSS) version 26 (IBM Corp, Armonk, NY, USA). Features were defined as the frequency distribution, mean, and standard deviation (SD) of the demographic information, and the TPB model factors were computed to examine the prevalence and influencing factors of Esports participation among Hong Kong university students. Content validity and construct validity were examined to determine the reliability of the questionnaire. Cronbach’s alpha coefficient was computed for the reliability analysis, where values greater than 0.70 are deemed to demonstrate the adequacy of the items’ internal consistency [28].

Exploratory factor analysis (EFA) is a statistical method that increases the reliability of a scale by identifying inappropriate items that can be removed. The factorial validity of the questionnaire was determined using the Kaiser–Meyer–Olkin (KMO) value and Bartlett’s test. A KMO value of at least 0.07 indicates an adequate, factorial, and valid questionnaire. For the construct validity of the questionnaire, confirmatory factor analysis (CFA) was evaluated using LISREL (Version 10.3, Scientific Software International, Inc). Other than factor loadings of each item accepted for values of 0.4 or above, the following fit indices for assessing the goodness of fit of the models were used for determination: values of χ^2^ ranging from 2 to 5, Comparative Fit Index (CFI) [26] and Non-Normed Fit Index (NNFI) rated as 0.90 or above [26]; standardized root mean square residual (SRMR) value of 0.08 or below [26]; root mean square error of approximation (RMSEA) value of 0.05 or below, and a 90% confidence interval that contains this value were considered the criteria of good fit [29].

### 3.2. Results

#### Demographics

In the current study, 25 secondary schools located in 11 Hong Kong districts (New Territories and Hong Kong Island) agreed to participate in this study. A total of 1576 students completed web-based and hardcopy questionnaires. After data screening consisting of excluding missing data and outliers, valid data for 1567 participants (male = 533; female = 615) were included in the study. Among the secondary school students, 48 of them had participated in Esports in the last six months.

Similarly, 1525 students at 10 UGC-funded universities completed the web-based questionnaires. After data screening, 951 university students underwent further data analysis. Among the 951 students (male = 490; female = 255), 56 had participated in Esports in the past six months. Most of them were degree students (60.5%) studying in Kowloon City (78.2%), and nearly 27.3% of students reported their household income as HKD 30,000 or higher. The participants’ demographics are presented in Table 1.

### 3.3. Explorative Factor Analysis

#### 3.3.1. Direct Measures

The diagnostic tests for all scales demonstrated adequacy of the exploratory factor analysis. For all scales, the Kaiser–Meyer–Olkin (KMO) value was over 0.8, and Bartlett’s test of sphericity was statistically significant. Moreover, all Cronbach’s *α* values were above 0.8, indicating satisfactory internal consistency. The EFA results for Intention, Attitude, Subjective Norms, and Perceived Behavioral Control yielded one factor, which accounted for 70–89% of the variance. The EFA results and the percentage range of variance between secondary school and university students were similar. Hence, all direct measures scales contained only one factor, and no items were deleted in the EFA stage.

#### 3.3.2. Indirect Measures

##### Behavioral Beliefs

In the Behavioral Beliefs items (Items 1–19), the EFA results for both secondary schools and universities indicated a three-factor solution, with two of the factors’ eigenvalues greater than 2.0, accounting for 63.2% and 57.1% of the variance, respectively. Item loading ranged from 0.7 to 0.87. In both samples, Items 8 and 9 were deleted upon the factor solution because of their low factor loadings (0.08 and 0.51). Item 3 was also deleted from the university sample. Item 3 belonged to Factor 1 in the secondary school sample and to Factor 2 in the university student sample. However, the content of item 3, which indicated “Interested in Esports would make me participate in Esports in the coming six months”, does not fit the factor of “Negative outcomes”. Therefore, Item 3 was deleted when examining the university sample.

As a result, in the secondary school student sample, Factor 1 consisted of 10 items representing the “Positive Outcomes” of Esports, while Factor 2 consisted of 5 items representing the “Negative Outcomes”. In the university student sample, Factor 1 consisted of 9 items representing the “Positive Outcomes” and the 5 items in Factor 2 representing the “Negative Outcomes” of Esports.

##### Normative Beliefs

Normative Beliefs consisted of 12 items, and the EFA results for both secondary schools and universities indicated two factors. However, only in the university data were two of the factors’ eigenvalues greater than 2.0, accounting for 64.4% of the variance. Item 10 was also removed from the university sample analysis due to low factor loading (0.27), while the secondary school students’ data demonstrated a factor eigenvalue of 1.99 for the second factor, accounting for 76.1% of the variance. Despite this, the 12 items were divided into two factors by item content. As a result, both samples indicated two factors, with Factor 1 consisting of eight items representing the “Significant Others” affecting Esports, while Factor 2 consisted of 3–4 items representing the “Personal Characteristics” of a person who would participate in sports.

##### Control Beliefs

Control Beliefs consisted of 19 items, and the EFA results for the secondary school and university data indicated four and five factors, respectively. However, only two of the factors’ eigenvalues were greater than 2.0, and the third factor’s eigenvalue of both samples was at 1.60, accounting for 65% and 59.78% of the variance, respectively. Nevertheless, three factors were indicated with regard to item content. Therefore, both samples indicated that Factor 1 consisted of 10 items representing the “Barriers” to participating in Esports, Factor 2 consisted of 3 items representing the “Facilitators” of participating in Esports, and Factor 3 consisted of 6 items representing the “Policy” related to sports participation.

### 3.4. Confirmatory Factor Analysis

#### 3.4.1. Direct Measures

##### Intention

In the TPB intention model, the secondary school sample showed perfect goodness-of-fit indices after the removal of Item 4 due to the high standardized residuals. Table 2 and Table 3 show the results of the reliability and CFA for all the individual measurement models of the current study.

The university sample achieved acceptable goodness of fit, with χ^2^(6.8/1) = 6.8, CFI = 0.99, NNFI = 0.99, SRMR = 0.003, RMSEA = 0.078 (90% CI = 0.031–0.14), after the covariance was added between “want to” and “plan to” participate in Esports.

##### Attitude

In the secondary school sample, no changes were made within the four attitude items, yielding satisfactory goodness of fit indices, with χ^2^(21.97/2) = 10.98, CFI = 0.994, NNFI = 0.0994, SRMR = 0.012, RMSEA = 0.093 (90% CI = 0.062–0.13). However, in the university sample, Item 1 “whether it is harmful/beneficial”, Item 2 “whether it is bad/good”, and Item 3 “whether it is unpleasurable or pleasurable” were correlated, in order to achieve a perfect goodness of fit.

##### Subjective Norms

In the secondary school sample, the initial model showed inadequate model fit, with χ^2^(36.84/2) = 18.42, CFI = 0.99, NNFI = 0.99, SRMR = 0.0056, RMSEA = 0.0056 (90% CI = 0.089–0.158). Item 2 “Participating in Esports is expected of me” was deleted due to the high residuals and the irrelevant content for secondary school students, after which excellent goodness of fit indices were obtained. No changes were made in the initial model for the university sample, yielding adequate goodness of fit indices, with χ^2^(4.38/2) = 2.19, CFI = 0.99, NNFI = 0.99, SRMR = 0.0037, RMSEA = 0.035 (90% CI = 0.00–0.082).

##### Perceived Behavioral Control

In the initial model of Perceived Behavioral Control, both samples showed inadequate model fit, with χ^2^(1006.37/2) = 503.18, CFI = 0.718, NNFI = 0.718, SRMR = 0.153, RMSEA = 0.657 (90% CI = 0.623–0.691) and χ^2^(1155.68/2) = 577.84, CFI = 0.58, NNFI = 0.58, SRMR = 0.238, RMSEA = 0.779 (90% CI = 0.741–0.817), respectively. Consequently, changes were made in the initial model of both secondary and university students sample, by allowing covariance between Item 3, “Able to control my rights to participate in Esports”, and Item 4, “I’m the one who decides whether I could participate in Esports”. Additionally, Item 1 was removed from the university sample because of the low factor loading of 0.28, yielding good model fit in both samples, with χ^2^(2.37/1) = 2.37, CFI = 1.00, NNFI = 0.099, SRMR = 0.0032, RMSEA = 0.034 (90% CI = 0.00–0.093) and χ^2^(4.38/2) = 2.19, CFI = 0.99, NNFI = 0.99, SRMR = 0.0042, RMSEA = 0.086 (90% CI = 0.00–0.106), respectively.

##### Behavioral Beliefs

In the initial model of behavioral beliefs, neither sample demonstrated a good model fit, with χ^2^(1763.53/103) = 17.1, CFI = 0. 890, NNFI = 0.884, SRMR = 0.0676, RMSEA = 0.118 (90% CI = 0.113–0.123) and χ^2^(981.71/103) = 9.5, CFI = 0.838, NNFI = 0.861, SRMR = 0.0728, RMSEA = 0.105 (90% CI = 0.095–0.112), respectively. The two samples demonstrated different results for the initial model modification. In the secondary school students sample, Items 1, 2, and 10 were removed due to high standardized residuals (from 24 to 69) and irrelevant content for secondary school students. For instance, Item 10 was “Participating in Esports would affect my income level”, for which secondary school students would have less considered an issue in their life. Moreover, based on the modification indices, the items for “Social Ability”, “Teamwork Ability”, “Community Skills”, “Self-Affirmation”, “Being Persistent”, “Achieving Goals”, “Time-Consuming”, and “Affects Academic” were correlated, yielding good model fit with χ^2^(499.54/71 = 7.03, CFI = 0.97, NNFI = 0.97, SRMR = 0.051, RMSEA = 0.068 (90% CI = 0.067–0.073). On the other hand, in the university student sample, no changes were made after the EFA results, while covariances were suggested between “Social Ability”, “Teamwork Ability”, “Community Skills”, “Setting Life Goals”, and “Life Feels Fulfilled”, yielding good model fit with χ^2^(430.14/99) = 4.34, CFI = 0.926, NNFI = 0.939, SRMR = 0.034, RMSEA = 0.066 (90% CI = 0.059–0.073).

##### Normative Beliefs

Both samples generated low goodness of fit in their initial Normative Beliefs models, with χ^2^(715.19/53) = 13.4, CFI = 0.928, NNFI = 0. 942, SRMR = 0. 0606, RMSEA = 0.104 (90% CI = 0.096–0.11) and χ^2^(296.63/43) = 8.9, CFI = 0.951, NNFI = 0.943, SRMR = 0.0593, RM SEA = 0.088 (90% CI = 0.078–0.097). Similarly, the secondary school and university students’ samples demonstrated different initial model modifications. In the secondary school sample, Items 8 and 10 were removed because of the high standardized residuals (at 140). Items that indicated “Peers’ Perceptions”, “Senior Peers’ Perceptions”, “Parents’ Perceptions”, “Teachers’ Perceptions”, “Perceptions of Peers who Did Not Play Esports”, “Close Friends’/Partners’ Perceptions”, and “Public Perceptions” were correlated, yielding good model fit with χ^2^(131.64/29) = 4.5, CFI = 0.99, NNFI = 0.99, SRMR = 0.055, RMSEA = 0.068 (90% CI = 0.067–0.074). On the other hand, only Item 8 was removed from the university sample, and items that indicated “Peers’ Perceptions”, “Senior Peers’ Perceptions”, “Teachers’ Perceptions”, “Public Perceptions”, “Perceptions of Peers who Did Not Play Esports”, and “Close Friends’/Partners’ Perceptions” were correlated. The CFA results showed good model fit with χ^2^(178.36/39) = 4.5, CFI = 0.973, NNFI = 0.966, SRMR = 0.0566, RMSEA = 0.068 (90% CI = 0.062–0.082).

##### Control Beliefs

Neither sample generated good model fit in the initial Control Beliefs model, with χ^2^(2049.94/116) = 17.6, CFI = 0.865, NNFI = 0.858, SRMSR = 0.079, RMSEA = 0.120 (90% CI = 0.115–0.124), and χ^2^(1617.90/132) = 12.2, CFI = 0.774, NNFI = 0.805, SRMR = 0.089, RMSEA = 0.121 (90% CI = 0.116–0.126). The initial Control Beliefs model consisted of three factors, and the secondary school and university student samples reacted differently to the three factors. In the secondary school student sample, Items 7 and 14 were removed because of the high standardized residuals (at 169.8) and irrelevancy. Moreover, according to the modification indices, items that indicated “Affecting Academics”, “Time Management Issues”, “Insufficient Esports Skills”, “Insufficient Physical Capability”, “Affecting Future Prospects”, “Gender Differences in Esports Skills”, “Insufficient Community Esports Promotion”, and “Insufficient Resources for Supporting Esports” were correlated. In addition, it showed adequate goodness of fit indices with χ^2^(483.65/94) = 5.1, CFI = 0.97, NNFI = 0.96, SRMR = 0.046, RMSEA = 0.059 (90% CI = 0.046–0.065). Compared to that of the university student sample, Item 9 was removed due to high measurement error, and only Item 7 was removed due to the high standardized residual. Nonetheless, more covariance between items was identified, other than those mentioned by the secondary school student sample, it also included “Insufficient Number of Team Members”, “Lack of Understanding/Chemistry among Teammates”, “Affects Salary/Income”, “Insufficient Esports-Related Education and Training”, and “Insufficient Systematic Competition/League Competition”. Finally, it showed adequate goodness of fit indices with χ^2^(428.89/94) = 4.5, CFI = 0.95, NNFI = 0.94, SRMR = 0.0702, RMSEA = 0.0682 (90% CI = 0.066–0.078).

## 4. Discussion

The current study aimed to develop and examine the psychometric properties of the ESPQ based on the TPB approach. Study 1 successfully facilitated the development of the item pool, and the results from Study 2 revealed that the TPB Esports Intention Questionnaire was reliable and valid in examining Esports participation intention among both Hong Kong secondary and university students. It is worth noting that there were comparable differences between secondary school and university students in the finalized items due to the different living status. For example, Esports in “Affecting Income” or “Facilitating the Achievement of Life Goals” had a higher factor loading in university than secondary school students. However, the questionnaire was applicable to both age groups.

In fact, there are existing questionnaires related to Esports but in the field of gaming consumption. These questionnaires were mainly developed based on gratification theory, which investigates why and how individuals actively seek Esports media consumption, in other words, viewing Esports tournament, broadcasting Esports competition and engaging in viewing platforms, yet not on the attitudes and intention in participating in Esports’ gaming or competition. Based on earlier research [30,31,32], the Video Gaming Consumption Scale involved six dimensions, including arousal, challenge, competition, diversion, fantasy, and social interaction, to which eight items were added [31] to form the Esports Consumption Motives dimension, which includes Identification with Sport, Entertainment, Sport Knowledge Application, Design/Graphics, Pass Time, Control, Skill Building for Playing Actual Sport, Permanence, and Peer Pressure. This scale showed values of Cronbach’s alpha ranging from 0.63 to 0.87 [31] and 0.8 to 0.89 [32].

In comparison to our scales, factors that are similar included control (to behavior control of individuals participating in Esports), peer pressure (corresponding to subjective norms or significant others affecting individuals ‘Esports’ participation or intention), skill building for playing actual sport (involved in the behavioral beliefs toward sports, such as physical skill [Q13.4] and mental skills, i.e., concentration judgment, communication skills, and teamwork [Q13.6–9]), social interaction (in line with the behavioral beliefs toward Esports in developing social skills Q13.5), and diversion (indicated in the behavioral beliefs item, able to relieve stress while engaging in Esports [Q13.11]). However, the present Esports Intention Questionnaire not only involved items that measured motives of those who were already engaged in or consumed sports, but also included items regarding demotivation or deleterious factors that prevent individuals from consuming Esports or that reduce individual intentions to engage in Esports. Additionally, the TPB model has been applied to different sports-related intention investigations besides gamification. Hence, the TPB Esports Intention Questionnaire is expected to be a tool that could provide a more comprehensive investigation of Esports intention and participation from both pro and con perspectives. Nonetheless, the current psychometric properties examination has indicated room for improvement, for instance, by involving test–retest reliability, as well as a separate sample for exploratory factor analysis. However, it worth noting that the questionnaire was developed based on the social context of Hong Kong, in which the applicability of the questionnaire among other Asian countries is uncertain, and this might also fluctuate based on the development of Esports among the general public of that county. Moreover, given that older adults Esports is seen to be emerging in Hong Kong, the current questionnaire’s generalizability is considered as narrow, thus a new TPB Esports Intention Questionnaire for older adults should be tailor made in the future.

## 5. Conclusions

To conclude, the newly designed TPB Esports Intention Questionnaire was found to be reliable and valid in revealing the level of intentions as well as the factors affecting Hong Kong students’ participation in Esports competitions. With the current questionnaire, an in-depth investigation of Hong Kong students’ Esports participation, as well as the potential relationship model between factors, could be examined using the theory of planned behavior approach in the near future. Moreover, with the validated questionnaire, a further investigation using the questionnaire to compare the Esports competition intention among different countries could be done in the future.

## Figures and Tables

**Table 1:** Sociodemographic characteristic of participants (Secondary School, *n* = 1164; University, *n* = 951).

Secondary School		
Age	*N*	%
Below 15 years	47	4.0%
15–19 years	1098	94.3%
20–25 years	12	1.0%
N/A	7	0.6%
Gender		
Male	533	45.8%
Female	615	52.8%
N/A		
Participated in Esports in last 6 months		
Yes	48	4.1%
No	1116	95.6%
Secondary grade		
Form 1	3	0.3%
Form 3	31	2.7%
Form 4	478	41.4%
Form 5	531	45.6%
Form 6	78	6.7%
School location		
Kowloon City	1	0.1%
Yuen Long	104	8.9%
Northern District	146	12.5%
Southern District	14	1.2%
Tuen Mun	110	9.5%
Sha Tin	304	26.1%
Shum Shui Po	69	5.9%
Wai Chai	40	3.4%
Tsuen Wan	1	0.1%
Kwai Chung	4	0.3%
Kwai Tsing	226	19.4%
Sai Kung	32	2.7%
Kwun Tong	112	9.6%
N/A	1	0.1%
House type		
Private house	281	24.1%
Home ownership scheme	155	13.3%
Public housing	607	52.1%
Others	84	7.2%
N/A	37	3.2%
Household income		
HKD 20,000 and below	375	32.2%
HKD 20,000–HKD 30,000	368	31.6%
HKD 30,000–HKD 40,000	189	16.2%
HKD 40,000–HKD 50,000	57	4.9%
HKD 50,000 and above	81	7.0%
N/A	94	8.1%

**Table 2:** Summary of subscales’ factorial validity and internal consistency.

Measurement Model		KMO	Cronbach’s *α*
**Direct Measures**			
Intention	Secondary	0.84	0.96
	University	0.83	0.95
Attitude	Secondary	0.82	0.96
	University	0.81	0.88
Subjective Norms	Secondary	0.87	0.88
	University	0.86	0.94
Perceived Behavioral Control	Secondary	0.63	0.92
	University	0.56	0.76
**Indirect Measures**			
Behavioral Beliefs	Secondary	0.93	0.88
	University	0.91	0.84
Normative Beliefs	Secondary	0.94	0.90
	University	0.90	0.86
Control Beliefs	Secondary	0.92	0.93
	University	/	0.91

Note. Table 2 summarizes the factorial validity (KMO value) and the internal consistency (Cronbach’s *α*) of each factor in the TPB model. Abbreviation: KMO = Kaiser–Meyer–Olkin value.

**Table 3:** Summary of goodness of fit of the measurement models.

Measurement Model		Chi-Squared Test		Indices			
		χ^2^/*df*	*p*	CFI	NNFI	SRMR	RMSEA (90% CI)
**Direct Measures**							
Intention	Secondary	Perfect Fit	<0.001	1.00	1.00	0.00	0.00
	University	6.61	<0.001	0.99	0.99	0.003	0.078 (0.031–0.138)
Attitude	Secondary	10.9	<0.001	0.99	0.99	0.012	0.093 (0.06–0.13)
	University	Perfect Fit	<0.001	1.00	1.00	0.00	0.00
Subjective Norms	Secondary	Perfect Fit	<0.001	1.00	1.00	0.00	0.00
	University	2.19	<0.001	0.99	0.99	0.004	0.0354 (0.00–0.082)
Perceived Behavioral Control	Secondary	2.37	<0.001	1.00	0.99	0.0032	0.034 (0.00–0.093)
	University	2.67	<0.001	0.99	0.99	0.0086	0.042 (0.00–0.106)
**Indirect Measures**							
Behavioral Beliefs	Secondary	7.03	<0.001	0.97	0.97	0.052	0.068 (0.069–0.074)
	University	4.34	<0.001	0.93	0.926	0.064	0.066 (0.059–0.073)
Normative Beliefs	Secondary	4.5	<0.001	0.99	0.99	0.03	0.055 (0.046–0.065)
	University	4.5	<0.001	0.97	0.902	0.057	0.072 (0.062–0.082)
Control Beliefs	Secondary	5.1	<0.001	0.97	0.96	0.046	0.06 (0.055–0.065)
	University	4.5	<0.001	0.91	0.913	0.07	0.072 (0.066–0.078)

Note. Table 3 summarizes all the goodness of fit indices of each factor in the TPB model, showing all factor/subscales in measuring the TBP of Esports participation are reliable and valid. Abbreviations: CFI = comparative fit index; NNFI = non-normed fit index; SRMR = standardized root mean square residual; RMSEA = root mean square error of approximation; *p* = *p*-value; χ^2^ = chi-square; *df* = degrees of freedom.

## Data Availability

The data presented in this study are available on request from the corresponding author.

## Supplementary Materials

The following are available online at https://www.mdpi.com/article/10.3390/ijerph182312653/s1, Supplementary Document S1: The Theory of Planned Behavior-Based Esports Intention Questionnaire.
