# Supplementary material for: Assessing Esports Participation Intention: The Development and Psychometric Properties of the Theory of Planned Behavior-Based Esports Intention Questionnaire (TPB-Esport-Q)
Source: Int J Environ Res Public Health. 2021 Nov 30;18(23):12653. doi: 10.3390/ijerph182312653 (PMC8656513; doi:10.3390/ijerph182312653)
Supplement: Supplementary file 1 [file ijerph-18-12653-s001.zip › ijerph-1375341-supplementary.pdf]

Supplementary Document S1

Hong Kong Baptist University

The Theory of Planned Behavior-Based Esports Intention Questionnaire (TPB-Esport-Q)

Part I: Esports Competition Participation Level

In the past six months, have you participated in any Esports competitions? YES/NO

A. If you answered [no], please skip to Parts II and III and complete all questions.

B. If you answered [Yes], please answer the following questions, and all questions in Parts II and III.

1. In the past six months, how many Esports competitions have you participated in?
2. On average, how much time (hours) do you spend each month in Esports competitions?
3. How much time (hours) do you spend on average in Esports training each day?
4. In the past 6 months, what was your favorite game? (Can be more than one)

☐ League of legends	☐ PUBG	☐ Rainbow 6
☐ Overwatch	☐ Fortnite	☐ CS:GO
☐ Dota2	☐ Street fighter V	☐ Others : ____________
☐ Starcraft	☐ Rocket League	

5. What equipment do you use in Esports competitions? (May be more than one)

☐ Console ☐ Computer ☐ Mobile phone ☐ Other : ____________

6. Do you often participate in Esports with your friends? Yes ☐/ No
7. Do you feel uncomfortable because you cannot expect to participate in Esports competitions? Yes ☐ / No ☐
8. Do your parents know that you are involved in Esports? Yes ☐ / No ☐

If yes, do they support your participation in Esports? Yes ☐ / No

Part II: Behavioral Status of the Theory of Planned Behavior (For secondary and university students)

Intentions for Participating in an Esports Competition

The following describes your intentions for participating in Esports in the next six months.

		Strongly disagree	Disagree	No opinion	Agree	Strongly agree
1.	I will participate in Esports competitions in the next six months.	1	2	3	4	5
2.	In the next six months, I expect to participate in Esports competitions.	1	2	3	4	5
3.	In the next six months, I would like to participate in an Esports competition.	1	2	3	4	5
4.	In the next six months, I plan to participate in Esports competitions.	1	2	3	4	5

Attitude towards participating in Esports competitions

The following describes your evaluation of your participation in e-sports in the next six months.

Do you think your participation in e-sports competitions in the next six months will be…

1.	Very Harmful	Harmful	No Opinion	Beneficial	Very Beneficial
2.	Very Bad	Bad	No Opinion	Good	Very Good
3.	Very Unpleasant	Unpleasant	No Opinion	Pleasant	Very Pleasant
4.	Very Unhelpful	Unhelpful	No Opinion	Useful	Very Useful

Subjective Norms for Esports participation

The following describes the impact of a person or group you value on your participation in Esports in the next six months.

		Strongly disagree	Disagree	No opinion	Agree	Strongly agree
1.	Many people or groups I value think I should be competing in Esports in the next six months.	1	2	3	4	5
2.	It is expected that I will be competing in Esports in the next six months.	1	2	3	4	5
3.	Crowd pressure is driving me to compete in Esports in the next six months.	1	2	3	4	5
4.	A person or group I value wants me to compete in Esports in the next six months.	1	2	3	4	5

Perceived behavioral control of participation in Esports competitions

The following describes how easy or difficult it will be for you to participate in e-sports competitions in the next six months.

		Strongly disagree	Disagree	No opinion	Agree	Strongly agree
1.	I believe I will be able to participate in e-competitions in the next six months.	1	2	3	4	5
2.	It will be easy for me to compete in e-sports in the next six months.	1	2	3	4	5

3.	I have full control over my decision to participate in e-sports in the next six months.	1	2	3	4	5
4.	It is entirely up to me to decide whether or not I will participate in e-sports in the next six months.	1	2	3	4	5

Behavioral beliefs about participating in Esports

The following is a description of the benefits and drawbacks of your participation in Esports in the next six months. Please read carefully and circle the most appropriate answer.

1	a	My participation in e-sports in the next six months will help define my life goals.	Strongly disagree	Disagree	No opinion	Agree	Strongly agree
	b	Setting goals in life for me is	Very Bad	Bad	No opinion	Good	Very good
2	a	I will feel fulfilled by competing in e-sports in the next six months.	Strongly disagree	Disagree	No opinion	Agree	Strongly agree
	b	Feeling fulfilled for me is	Very Bad	Bad	No opinion	Good	Very good
3	a	My involvement in e-sports will be my hobby for the next six months.	Strongly disagree	Disagree	No opinion	Agree	Strongly agree
	b	My interest for me is	Very Bad	Bad	No opinion	Good	Very good
4	a	My participation in e-sports for the next six months will promote physical fitness/physical ability (physical fitness, hand-eye coordination,	Strongly disagree	Disagree	No opinion	Agree	Strongly agree

		mental reactivity).					
	b	Promoting physical health/physical ability (physical fitness, hand-eye coordination, mental reactivity) is	Very Bad	Bad	No opinion	Good	Very good
5	a	My participation in e-sports in the next six months will increase my social skills (meeting new people, improving friendships).	Strongly disagree	Disagree	No opinion	Agree	Strongly agree
	b	Increasing social skills (meeting new people, improving friendships) is	Very Bad	Bad	No opinion	Good	Very good
6	a	My participation in e-sports in the next six months will increase my teamwork skills.	Strongly disagree	Disagree	No opinion	Agree	Strongly agree
	b	Increasing teamwork skills for me is	Very Bad	Bad	No opinion	Good	Very good
7	a	My participation in e-sports competitions in the next six months will increase my communication skills.	Strongly disagree	Disagree	No opinion	Agree	Strongly agree
	b	Enhanced communication skills for me would be	Very Bad	Bad	No opinion	Good	Very good
8	a	My participation in e-sports in the next six months will reduce my judgment.	Strongly disagree	Disagree	No opinion	Agree	Strongly agree
	b	Decreased judgment for me is	Very Bad	Bad	No opinion	Good	Very good

9	a	My participation in e-sports for the next six months will reduce my ability to concentrate.	Strongly disagree	Disagree	No opinion	Agree	Strongly agree
	b	Decreased focus for me is	Very Bad	Bad	No opinion	Good	Very good
10	a	My participation in e-sports for the next six months will help increase my income.	Strongly disagree	Disagree	No opinion	Agree	Strongly agree
	b	Helping to increase my income would be	Very Bad	Bad	No opinion	Good	Very good
11	a	My participation in e-sports competitions in the next six months will help reduce stress.	Strongly disagree	Disagree	No opinion	Agree	Strongly agree
	b	Helps reduce stress for me	Very Bad	Bad	No opinion	Good	Very good
12	a	My participation in e-sports competitions in the next six months will increase my self-confidence.	Strongly disagree	Disagree	No opinion	Agree	Strongly agree
	b	Improving self-affirmation for me is	Very Bad	Bad	No opinion	Good	Very good
13	a	My participation in e-sports in the next six months will hinder my education.	Strongly disagree	Disagree	No opinion	Agree	Strongly agree
	b	Hindering my education is	Very Bad	Bad	No opinion	Good	Very good
14	a	My participation in e-sports competitions in the next six months will be time-consuming.	Strongly disagree	Disagree	No opinion	Agree	Strongly agree
	b	The fee time for me is	Very Bad	Bad	No opinion	Good	Very good
15	a	My participation in e-	Strongly	Disagree	No	Agree	Strongly

		sports competitions in the next six months will make my income unstable.	disagree		opinion		agree
	b	Unstable income for me is	Very Bad	Bad	No opinion	Good	Very good
16	a	My participation in e-sports for the next six months will result in injuries (cervical, vision, etc.).	Strongly disagree	Disagree	No opinion	Agree	Strongly agree
	b	Injuries (cervical, vision, etc.) to me are	Very Bad	Bad	No opinion	Good	Very good
17	a	My participation in e-sports in the next six months will cause family conflict.	Strongly disagree	Disagree	No opinion	Agree	Strongly agree
	b	Causing family conflict for me is	Very Bad	Bad	No opinion	Good	Very good
18	a	My participation in e-sports for the next six months will build perseverance/will.	Strongly disagree	Disagree	No opinion	Agree	Strongly agree
	b	Building perseverance/willpower for me is	Very Bad	Bad	No opinion	Good	Very good
19	a	My participation in e-sports in the next six months will help achieve my goals	Strongly disagree	Disagree	No opinion	Agree	Strongly agree
	b	Helping to achieve my goals would be	Very Bad	Bad	No opinion	Good	Very good

Normative beliefs about participating in Esports competitions

The following is a description of the social pressure or criteria that you will feel in the next six months as a result of participating in e-sports. Please read them carefully and circle the most appropriate answer.

1	a	Do my parents and family think I should compete in e-sports in the next six months?	Strongly disagree	Disagree	No opinion	Agree	Strongly agree
	b	I want to do what my parents and family think I should do?	Strongly disagree	Disagree	No opinion	Agree	Strongly agree
2	a	My peers who are involved in e-sports think I should compete in e-sports in the next six months?	Strongly disagree	Disagree	No opinion	Agree	Strongly agree
	b	I want to do what my peers in esports think I should do.	Strongly disagree	Disagree	No opinion	Agree	Strongly agree
3	a	Do my peers who do not participate in e-sports think I should participate in e-sports in the next six months?	Strongly disagree	Disagree	No opinion	Agree	Strongly agree
	b	I want to do what my non-competitive peers think I should do.	Strongly disagree	Disagree	No opinion	Agree	Strongly agree
4	a	Should my peers who are involved in e-sports think I should compete in e-sports in the next six months?	Strongly disagree	Disagree	No opinion	Agree	Strongly agree
	b	I want to do what my esports seniors think I should do.	Strongly disagree	Disagree	No opinion	Agree	Strongly agree
5	a	Does my teacher think I should compete in e-sports in the next six months?	Strongly disagree	Disagree	No opinion	Agree	Strongly agree
	b	I want to do what my	Strongly	Disagree	No	Agree	Strongly

		teacher thinks I should do.	disagree		opinion		agree
6	a	Does my partner/best friend think I should compete in e-sports in the next six months?	Strongly disagree	Disagree	No opinion	Agree	Strongly agree
	b	I want to do what my partner/best friend thinks I should do.	Strongly disagree	Disagree	No opinion	Agree	Strongly agree
7	a	Do people think I should compete in e-sports in the next six months?	Strongly disagree	Disagree	No opinion	Agree	Strongly agree
	b	I want to do what the public thinks I should do.	Strongly disagree	Disagree	No opinion	Agree	Strongly agree
8	a	All participants who are hopeful about the development of e-sports will be competing in e-sports in the next six months.	Strongly disagree	Disagree	No opinion	Agree	Strongly agree
	b	I want to be a hopeful person when it comes to competing in e-sports.	Strongly disagree	Disagree	No opinion	Agree	Strongly agree
9	a	Mentally competent e-sports participants will participate in e-sports competitions in the next six months.	Strongly disagree	Disagree	No opinion	Agree	Strongly agree
	b	I want to be mentally competent when it comes to competing in e-sports.	Strongly disagree	Disagree	No opinion	Agree	Strongly agree
10	a	A strong and resilient person in the e-sports community will be	Strongly disagree	Disagree	No opinion	Agree	Strongly agree

		competing in e-sports in the next six months.					
	b	When it comes to competing in e-sports, I want to be a person of perseverance.	Strongly disagree	Disagree	No opinion	Agree	Strongly agree
11	a	Participants who have financial support will participate in e-sports for the next six months.	Strongly disagree	Disagree	No opinion	Agree	Strongly agree
	b	I would like to be a financially supported person when it comes to competing in e-sports.	Strongly disagree	Disagree	No opinion	Agree	Strongly agree
12	a	Highly competent e-sports players will participate in e-sports competitions in the next six months.	Strongly disagree	Disagree	No opinion	Agree	Strongly agree
	b	When it comes to competing in e-sports, I would like to be a high e-sports player.	Strongly disagree	Disagree	No opinion	Agree	Strongly agree

Control Beliefs about participating in Esports

The following describes the barriers and facilitators you will have in participating in e-sports in the next 6 months. Please read them carefully and circle the most appropriate number of words.

In the next six months, you expect to

			Strongly disagree	Disagree	No opinion	Agree	Strongly agree
1	a	Academics will be hindered.	1	2	3	4	5
	b	Academic hindrance	1	2	3	4	5

		will make it easier for me not to compete in e-sports for the next six months.					
2	a	Poor time management (rework, training, academics).	1	2	3	4	5
	b	Poor time management (back to work, training, school) makes me vulnerable to not competing in e-sports for the next six months.	1	2	3	4	5
3	a	Inadequate e-sports skills.	1	2	3	4	5
	b	Inadequate e-sports skills make me vulnerable to not competing in e-sports in the next six months.	1	2	3	4	5
4	a	Inadequate physical abilities (e.g., reflexes).	1	2	3	4	5
	b	Inadequate physical skills (e.g., reflexes) make me vulnerable to not competing in e-sports in the next six months.	1	2	3	4	5
5	a	There will be income problems at work.	1	2	3	4	5
	b	Having a work income problem makes me vulnerable to not competing in e-sports in the next six months.	1	2	3	4	5
6	a	There will be problems	1	2	3	4	5

		with my future.					
	b	Having a future problem makes me vulnerable to not competing in e-sports for the next six months.	1	2	3	4	5
7	a	The development of e-sports in Hong Kong is not good.	1	2	3	4	5
	b	Poor e-sports development in Hong Kong makes me vulnerable to not competing in e-sports in the next six months.	1	2	3	4	5
8	a	There will be a gender gap in e-sports skills.	1	2	3	4	5
	b	The gender gap in e-sports skills will make me vulnerable to not competing in e-sports in the next six months.	1	2	3	4	5
9	a	There are not enough people on the team.	1	2	3	4	5
	b	Insufficient team size makes me vulnerable to not competing in e-sports in the next six months.	1	2	3	4	5
10	a	Lack of team chemistry.	1	2	3	4	5
	b	Lack of team chemistry makes me vulnerable to not competing in e-sports in the next six months.	1	2	3	4	5
11	a	Get a sense of	1	2	3	4	5

		accomplishment.					
	b	Gaining a sense of accomplishment makes me more likely to compete in e-sports in the next six months.	1	2	3	4	5
12	a	Be able to express myself.	1	2	3	4	5
	b	Being able to express myself makes it easier for me to participate in e-sports competitions in the next six months.	1	2	3	4	5
13	a	Will have peer support.	1	2	3	4	5
	b	Peer support will make it easy for me to compete in e-sports in the next six months.	1	2	3	4	5
14	a	The location of the e-sports venue is not convenient.	1	2	3	4	5
	b	The inconvenient location of the venue makes it easy for me not to participate in e-sports competitions in the next six months.	1	2	3	4	5
15	a	Inadequate eSports education and training.	1	2	3	4	5
	b	Inadequate e-sports education and training makes me vulnerable to not competing in e-sports in the next six months.	1	2	3	4	5
16	a	Insufficient publicity and promotion in the	1	2	3	4	5

		community.					
	b	Insufficient publicity and promotion in the community makes me vulnerable to not competing in e-sports in the next six months.	1	2	3	4	5
17	a	There is a lack of policy on e-sports development.	1	2	3	4	5
	b	Lack of eSports related development policy makes me vulnerable to not participating in eSports competitions in the next six months.	1	2	3	4	5
18	a	Lack of e-sports related competitions and leagues.	1	2	3	4	5
	b	The lack of eSports related tournaments and leagues makes me vulnerable to not participating in eSports tournaments in the next six months.	1	2	3	4	5
19	a	Lack of esports resources (e.g., sponsorship, government resources).	1	2	3	4	5
	b	Lack of esports resources (e.g., sponsorship, government resources) makes me vulnerable to not competing in esports in the next six	1	2	3	4	5

months
